# Fine-Grained Semantics-Enhanced Graph Neural Network Model for Person-Job Fit

**DOI:** 10.3390/e27070703

**Published:** 2025-06-30

**Authors:** Xia Xue, Jingwen Wang, Bo Ma, Jing Ren, Wujie Zhang, Shuling Gao, Miao Tian, Yue Chang, Chunhong Wang, Hongyu Wang

**Affiliations:** 1 Maths and Information Technology School, Yuncheng University, Yuncheng 044000, China; ztyx_2023@163.com (J.W.);; 2 School of Foreign Languages, Northwest University, Xi’an 710127, China; shulinggao1998@163.com; 3 School of Computer Science and Technology, Xi’an University of Posts and Telecommunications, Xi’an 710121, China; hywang@xupt.edu.cn

**Keywords:** person-job fit, graph neural network, fine-grained semantics, cross-entropy

## Abstract

Online recruitment platforms are transforming talent acquisition paradigms, where a precise person-job fit plays a pivotal role in intelligent recruitment systems. However, current methodologies predominantly rely on coarse-grained semantic analysis, failing to address the textual structural dependencies and noise inherent in resumes and job descriptions. To bridge this gap, the novel fine-grained semantics-enhanced graph neural network for person-job fit (FSEGNN-PJF) framework is proposed. First, graph topologies are constructed by modeling word co-occurrence relationships through pointwise mutual information and sliding windows, followed by graph attention networks to learn graph structural semantics. Second, to mitigate textual noise and focus on critical features, a differential transformer and self-attention mechanism are introduced to semantically encode resumes and job requirements. Then, a novel fine-grained semantic matching strategy is designed, using the enhanced feature fusion strategy to fuse the semantic features of resumes and job positions. Extensive experiments on real-world recruitment datasets demonstrate the effectiveness and robustness of FSEGNN-PJF.

## 1. Introduction

Online recruitment platforms (e.g., LinkedIn, Zhipin, and Liepin) have emerged as mainstream tools for talent recruitment due to their efficiency [1]. As of July 2023, LinkedIn reported over 930 million global users across 200+ countries, underscoring its widespread adoption. However, this growth has exacerbated information overload, necessitating advanced methods to accurately align job requirements with candidate capabilities from massive datasets, a critical challenge in person-job fit research.

Early approaches framed person-job fit as a recommendation task [2,3]. For instance, Malinowski et al. [2] proposed a bilateral recommendation model via mining key characteristics such as candidate skills and educational backgrounds. While effective, such methods incur high costs and subjective biases due to their reliance on domain expertise. Subsequent studies shifted toward supervised text matching.

More recently, with the resurgence of deep learning, deep learning-based person-job fit methods have garnered significant attention from researchers. Zhu et al. [4] employed parallel convolutional neural networks (CNNs) to extract the features of resumes and job positions and measure similarity via cosine metrics. Qin et al. [1] integrated bidirectional long short-term memory (BiLSTM) with hierarchical attention to prioritize critical skills, while Shao et al. [5] utilized bidirectional encoder representations from transformers (BERT) and multi-head attention to explore internal and external interactions for multivariate attributes in job-resume matching. Despite the progress made by the aforementioned methodologies in the field of person-job fit, they are still confronted with several notable challenges. On the one hand, candidate resumes and job requirement descriptions often contain noisy information, and existing methods demonstrate limited capacity in filtering out such information, thereby undermining the matching performance. On the other hand, current approaches predominantly rely on coarse-grained semantics to match resumes with job requirements, impeding the comprehensive exploration of the full matching potential between the two parties.

To capture the deep semantic associations and textual structural information between job seekers’ resumes and job requirements, graph attention networks (GATs) were introduced. A differential attention mechanism is incorporated to mitigate the impact of noise, enabling more accurate semantic representations of both resumes and job requirements. The primary objective is to further capture the global dependencies within the texts of resumes and job requirements, focusing on the importance weights of different words. A self-attention mechanism is employed to semantically encode the resumes and job positions. To facilitate semantic matching between job seekers’ resumes and job requirements from a multi-granularity perspective, a fine-grained semantic matching strategy at the resume-job position level was devised. Inspired by the successful application of the Kronecker attention network (KAN) to capturing nonlinear relationships, we introduce it into the person-job matching task.

To this end, this paper proposes a framework termed fine-grained semantics-enhanced graph neural network for person-job fit. Inspired by the successful application of graph neural network (GNN)-based methods [6,7] in other tasks, a GNN is introduced into the person-job fit task. Specifically, initially, key features, including skills and experiences, are extracted from both resumes and job postings and defined as nodes within a graph structure. The edges connecting these nodes are established through pointwise mutual information (PMI) and a sliding window method, which effectively captures the contextual associations between job requirements and resume attributes. Subsequently, GATs are employed to learn the structural representations of the constructed graph. To further optimize the encoding process, a differential transformer (DIFF Transformer) is introduced. This model encodes both resume and job posting inputs and integrates self-attention mechanisms, thereby significantly enhancing the model’s ability to focus on critical features relevant to job-resume fit. Finally, a novel fine-grained semantic matching computation method is designed. This method leverages Kolmogorov-Arnold networks (KANs) to deeply fuse semantic features from applicants’ resumes and job requirements, thereby significantly improving person-job fit performance. The experimental results validate the effectiveness of the FSEGNN-PJF framework.

The principal contributions of this paper are as follows:

(1) An innovative graph construction methodology, grounded in the principles of co-occurrence windows and PMI, has been meticulously developed to construct graph representations for job seekers’ resumes and job requirement texts. The overarching objective of this approach is to conduct an in-depth exploration of the semantic structural interdependencies embedded within these texts. By leveraging GATs, the graph structures are encoded, facilitating the enhancement of node feature representations through the aggregation of pertinent neighborhood information.
(2) A sophisticated semantic encoding framework tailored to resumes and job requirements and integrating a hybrid attention mechanism is proposed. This framework is designed to adeptly capture the semantic dependencies between job seekers’ resumes and job requirements, dynamically allocate feature weights, and effectively filter out noise within data. Consequently, it significantly bolsters the semantic representations of both resumes and job requirements, thereby refining their semantic fidelity.
(3) A meticulously crafted fine-grained semantic matching computation methodology is devised which synergistically combines multi-granularity text similarity measurement strategies. A KAN is introduced to optimize the activation function expression and augment the fine-grained semantic representations inherent in job seekers’ resumes and job requirements, culminating in the attainment of highly precise person-job matching performance.
(4) Empirical validation showing the novel FSEGNN-PJF framework achieves state-of-the-art performance.

The rest of this paper is organized as follows. Section 2 reviews the recruitment analysis and text match literature. Section 3 formalizes the problem and details the FSEGNN-PJF framework. Section 4 presents the experimental set-up, baselines, evaluation metrics, overall performance, ablation study, and case study. Section 5 concludes this work.

## 2. Literature Review

The related work in this paper is categorized into two main areas: recruitment analysis and text matching.

### 2.1. Recruitment Analysis

Recruitment serves as the fundamental method for organizational talent acquisition, playing a pivotal role in enterprise success. As a critical task in talent management, effective recruitment strategies aim to optimize candidate-position matches, with empirical evidence demonstrating that employee-position mismatches cause significant turnover [8,9]. The burgeoning field of person-job fit research has emerged as a focal point in contemporary human resource analytics.

Several researchers have studied person-job fit from a performance prediction [10,11] perspective. In particular, the matching of recruitment positions with job seekers’ resumes has received great attention from researchers. Recommendation system paradigms have yielded notable contributions, including hybrid collaborative filtering techniques combining K-nearest neighbor algorithms with clustering approaches to enhance recommendation accuracy [12]. Bilateral recommendation methods have further advanced the field through separate modeling of candidates and job position preference relationships [2]. A job recommendation model based on gradient-boosted regression trees (GBRTs) and time factors incorporating GBRTs with neighborhood-based filtering demonstrated improved preference prediction through temporal feature extraction [13]. In the LinkedIn recommendation system, a generalized linear mixed model achieved scalable job recommendations through user- and item-level personalization [3].

Recently, researchers have studied the person-job fit task from another perspective. Deep learning technology has achieved impressive performance in the person-job fit task. A parallel CNN enabled semantic matching of resumes and position descriptions through shared latent space mapping and cosine similarity methods [4]. An ability-aware person-job fit model employing BiLSTM networks with multi-level hierarchical attention mechanisms introduced encoding representation and ability-aware representation learning of job seekers’ resumes and job requirements [1]. Feature fusion approaches combining a factorization machine-based neural network and CNN with long short-term memory network modeling effectively integrated explicit and implicit characteristics from historical recruitment data to extract the global features of resumes and job requirements [14]. Subsequent studies addressed critical limitations in preference modeling through gated recurrent unit-based frameworks incorporating historical interaction patterns [15], while reinforcement learning strategies introduced a dynamic matching optimization strategy considering the labor market fluctuations [16].

Current research frontiers focus on sophisticated semantic modeling through transformer architectures. The BERT framework has demonstrated superior contextual understanding in cross-domain matching tasks. Recent studies employed BERT to project attribute keys, values, and their sources from both resumes and job postings into a unified semantic space and then employed multi-head attention mechanisms to model intra-attribute and inter-attribute interactions across candidate resumes and position requirements [5]. This progression from traditional recommendation systems to deep semantic modeling reflects the field’s evolution toward sophisticated context-aware matching paradigms.

### 2.2. Text Matching

Text-based person-job matching constitutes a specialized text mining task fundamentally aligned with natural language processing (NLP) paradigms, particularly text classification [17]. As a core NLP task, text matching has witnessed substantial methodological evolution through deep learning advancements, with contemporary research predominantly leveraging deep learning technology for text matching.

Current methodologies bifurcate into representation-based and interaction-based paradigms. The former employs Siamese networks for semantic similarity computation through two-sentence encoding. Pioneering work includes deep structured semantic models (DSSMs) utilizing deep neural networks for semantic vector projection and cosine similarity measurement [18], and convolutional neural network architectures extracting lexical matching patterns from pretrained embeddings via convolution layers and pooling layers [19]. While effective in individual text representation, these approaches exhibit limitations in modeling cross-text interactions.

Interaction-based methods address this gap through explicit inter-sentence relationship modeling. Enhanced sequential inference frameworks combine BiLSTM-processed embeddings with soft attention mechanisms for high-order interaction extraction [20]. Iterative interaction modules with multi-perspective pooling layers enable progressive relational understanding between text pairs [21]. Graph-based relevance models introduce structural flexibility through term-level interaction and document-level relationship modeling, effectively addressing long-distance semantic matching challenges [22]. Advanced architectures integrate frame semantics with multi-level attention mechanisms, capturing contextualized frame elements and hierarchical semantic relationships [23]. Recent innovations employ keyword-centric attention strategies to simultaneously model word-level alignments and sentence-level semantic dependencies [24]. This paradigm shift from isolated representation learning to explicit interaction modeling has significantly advanced text matching precision in complex semantic environments.

## 3. Method

**Figure 1:**
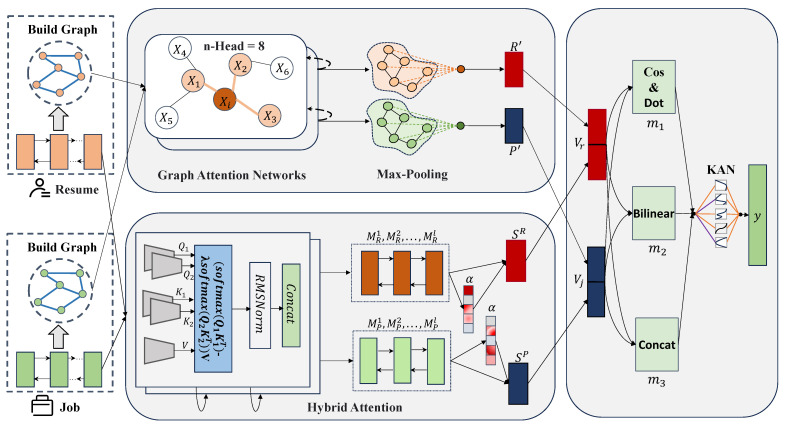
The architecture of the proposed FSEGNN-PJF framework.

The proposed fine-grained semantics enhanced graph neural network for person-job fit framework comprises three structured components: graph-based semantic encoding for resumes and job positions, hybrid attention mechanism-based semantic encoding for resumes and job positions, and person-job fit. Figure 1 illustrates the architectural of the proposed FSEGNN-PJF framework.

Subsequently, based on a resume and a job description, the workflow of the FSEGNN-PJF framework will be elaborated in detail. The framework utilizes the applicant’s resume (Resume) and the job requirements (Job) posted by the enterprise as inputs and outputs the prediction results for person-job fit.

Specifically, in the graph-based semantic encoding of resumes and job descriptions, firstly, text graph structures for both the applicant’s resume and the job requirements are constructed. The texts from the resume and the job description are preprocessed through tokenization and other necessary steps, yielding word sequences for the resume (“Asp”, “SQLServer”, “database”, “proficient in”, etc.) and for the job requirements (“database”, “programming language”, “JavaScript”, etc.). These word sequences serve as nodes in the graph. After that, the PMI values between nodes determining the weights of the edges are calculated, thereby highlighting the semantic association strengths among key skills (e.g., “SQLServer”, which is one of the “database programming languages”). This approach provides more effective structural information for the graph neural network. Subsequently, GATs are then employed to encode and learn from the constructed text graphs, aiming to capture the semantic structures within both the resume and job requirements.

In the semantic encoding module for resumes and job descriptions based on a hybrid attention mechanism, a differential attention encoder is primarily employed to encode both the resume and job requirements. This encoder is capable of effectively reducing the attention weights assigned to misspelled or noisy words, such as “jingxing”. Subsequently, a self-attention mechanism is utilized to further focus on the key skill features within both the resume and job requirements (“JavaScript”, “SQLServer”, “database”, etc.). Finally, considering that terms like “.Net platform” and “.Net framework” are highly similar in semantics but exhibit low similarity in their word vector representations, adversely affecting the matching results, a feature fusion strategy with fine-grained semantic enhancement is designed to effectively integrate the graph-based semantic encoding features and hybrid attention-based semantic encoding features, thereby determining the match between the applicant’s resume and the job posting.

### 3.1. Problem Definition

This paper aims to study the matching between job seekers’ resumes and job requirements in person-job fit tasks. Specifically, given a job seeker’s resume containing n skills, denoted as $R=\{r_{1},r_{2},r_{3},\ldots ,r_{n}\}$, each skill $r_{l}$ consists of s words, represented by $r_{l}=\left\{r_{l,1},r_{l,2},r_{l,3},\ldots ,r_{l,s}\right\}$. Similarly, a job posting contains *m* requirements, denoted as $P=\{p_{1},p_{2},p_{3},\ldots ,p_{m}\}$, with each job requirement $p_{j}$ comprising *v* words, represented by $p_{j}=\left\{p_{j,1},p_{j,2},p_{j,3},\ldots ,p_{j,v}\right\}$. A label y∈{0,1} is used to indicate the recruitment outcome, where y=1 signifies a successful match and y=0 indicates a mismatch.

Based on the above definitions, the person-job fit task can be formulated as learning a predictive model from the interaction records of existing job seekers’ resumes and job requirements. This model calculates the matching score between *R* and *P* and subsequently predicts the corresponding outcome label *y*.

### 3.2. Graph-Based Semantic Encoding for Resumes and Job Positions

Enterprises primarily rely on keyword-based matching of skills and experiences to identify suitable candidates. However, this approach struggles to accurately capture the deep semantic associations and textual structural information between job seekers’ resumes and job requirements. To address this issue, graph networks need to be introduced into the person-job matching task. The initial step in employing graph networks involves graph construction.

#### 3.2.1. Graph Construction Methods for Resumes and Job Positions

The resumes *R* and the job requirements *P* to be matched need to be transformed into graph structures. Next, taking resumes as an example, the specific way to construct a graph structure is as follows.

First, an empty undirected graph *G* is initialized. Each skill $r_{l}$ in the job seekers’ resume texts *R* is segmented into a word sequence. The word sequences of the resumes are traversed in order $r_{l}=\left\{r_{l,1},r_{l,2},r_{l,3},\ldots r_{l,s}\right\}$. If a word exists in the pretrained Word2Vec word embedding model, then its corresponding n–dimensional feature vector $h_{r_{l,s}}$ is obtained. In this context, the word vectors for all nodes in both the resume graph and the job graph are derived from the same pretrained Word2Vec model. The words are added as nodes to the graph *G*, with the corresponding word vector serving as the nodes’ representation.

Second, edges are constructed using a co-occurrence window approach. Specifically, for the segmented sequence of the job seekers’ resumes, edges are formed between words within the co-occurrence window range. The above steps are repeated until a complete graph structure is constructed.

Finally, PMI is utilized to quantify the semantic association strength between key features. Subsequently, a sliding window approach is employed to construct edges between nodes, with weights assigned to these edges to focus on the strong associative relationships among local features. The probability of each pair of connected nodes representing words co-occurring in the resumes in graph G is calculated and denoted as $P(w_{i},w_{j})$. The probability of each node’s word occurring individually is calculated as $P(w_{i})$ and $P(w_{j})$. The PMI between adjacent words is computed using the following formula:

(1) $$PMI\left(w_{i},w_{j}\right)=log\frac{P(w_{i},w_{j})}{P\left(w_{i}\right)P\left(w_{j}\right)}$$

where $w_{i}$ represents the word corresponding to the *i*th node in graph *G* and $w_{j}$ represents the word corresponding to the *j*th node. When the PMI is positive, a weighted edge is added between the nodes corresponding to the two words, with the weight being the PMI value.

The graph construction method for the job requirements *P* to be matched is similar to the above method for the job seekers’ resumes.

#### 3.2.2. Graph Attention Network-Based Semantic Encoding for Resumes and Job Positions

GATs [25], by stacking multiple layers of network structures, enable each node to dynamically attend to the features of its neighboring nodes. This approach adaptively learns the importance weights between nodes, effectively extracting and encoding key features from job seekers’ resumes and job requirements.

Taking job seekers’ resumes as an example, the resume graph is represented by R=(V,E), where *V* denotes the node sets and *E* denotes the edge sets. The similarity $e_{ij}$ between node *i* (denoted as $v_{i}$) and node *j* (denoted as $v_{j}$) via linear transformation and concatenation operation is calculated using the formula:

(2) $$e_{ij}^{\left(l\right)}=\sigma \left(a^{(l)T}\left[W^{\left(l\right)}h_{i}∥W^{\left(l\right)}h_{j}\right]\right)$$

where $h_{i}$ and $h_{j}$ are the linearly transformed features of $v_{i}$ and $v_{j}$, respectively, $W^{\left(l\right)}$ represents the trainable parameters of the *l*th attention head, $a^{(l)T}$ represents the parameters of the *l*th attention head, σ is the LeakyReLU activation function, and ‖ denotes the concatenation operation.

The softmax function is then applied to normalize the similarity $e_{ij}^{\left(l\right)}$ between $v_{i}$ and $v_{j}$, yielding the attention coefficient $\alpha_{ij}^{\left(l\right)}$. The formula is as follows:

(3) $$\alpha_{ij}^{\left(l\right)}=\frac{exp(e_{ij}^{\left(l\right)})}{\sum_{k\in N_{i}}exp\left(e_{ik}^{\left(l\right)}\right)}$$

where $N_{i}$ is the set of neighboring nodes of node *i*.

After constructing the resume graph, the features of each node’s neighboring nodes are weighted and summed using the attention coefficients. The feature representations from each attention head are then concatenated to obtain the node features $h_{i}^{1}$ of the resume. The formula for this is as follows:

(4) $$h_{i}^{1}=\underset{l=1}{\overset{L}{∥}}\sigma \left(\sum_{j\in N_{i}}\alpha_{ij}^{\left(l\right)}W^{\left(l\right)}h_{j}\right)$$

where *L* is the total number of attention heads, σ is the LeakyReLU activation function, and ‖ denotes vector concatenation.

Through the above method, a single GAT layer is obtained. In the experiments, a two-layer stacked GAT layer is employed, where each GAT layer updates and aggregates the node features to obtain refined node representations $h_{i}^{2}$.

Subsequently, global max pooling is used to refine and compress the information within the graph attention network structure. Specifically, for the neighborhood feature vectors of each node, the element-wise maximum value is selected as the node’s global feature representation. The formula for this is as follows:

(5) $$\;R^{\prime }=Maxpooling\left(h_{i}^{2}\right)$$

The semantic encoding method $P^{\prime }$ for the job requirements to be matched is similar to the above semantic encoding method for the job seekers’ resumes.

### 3.3. Hybrid Attention Mechanism-Based Semantic Encoding for Resumes and Job Positions

In this subsection, a semantics-focused hybrid attention mechanism encoding method is introduced to capture the semantics of job seekers’ resumes and enterprise recruitment requirements.

#### 3.3.1. Differential Attention Mechanism-Based Semantic Encoding for Resumes and Job Positions

Differential attention mechanism mitigates the impact of noise, enabling more accurate semantic representation of the content within job seekers’ resumes and job requirements. Taking job seekers’ resumes as an example, the resume semantic encoding method using the differential attention mechanism is as follows.

First, the pretrained word embedding matrix $W_{e}$ is used to map job seekers’ skills into a continuous vector space:

(6) $$W_{l,s}^{R}=W_{e}r_{l,s}$$

where $r_{l,s}$ denotes the *s*th word in the *l*th skill and $W_{l,s}^{R}$ represents the vector of the *s*th word within the skill $r_{l}$. It is crucial to emphasize that the word embedding matrix is the shared $W_{e}$ between enterprise recruitment requirements and job seekers’ resumes.

Subsequently, the resumes’ representation of the job seekers $X_{R}$ is obtained, and the calculation formula is as follows:

(7) $$X_{R}=\left[W_{1,1:s}^{R},W_{2,1:s}^{R},\cdots ,W_{n,1:s}^{R}\right]$$

where $W_{n,1:s}^{R}$ represents the embedded representation of the *n*th skill. For example, for 1,1:*s*, 1 represents the first skill, and 1:*s* refers to the first to *s* words in the first skill. The rest follow similarly.

Next, the multi-head differential attention mechanism from the differential transformer (Diff-Transforms) [26] is introduced to encode the job seekers’ resume. To remove noise from the attention scores, the difference between a pair of softmax functions is used to eliminate noise.

Specifically, given the embedded representation of the job seekers’ resumes $X_{R}$, it is projected into a query, key, and value. By multiplying with the weight matrices of the query, key, and value ($W_{i}^{Q}$, $W_{i}^{K}$, and $W_{i}^{V}$, respectively), the representations of the query $Q_{1}$ and $Q_{2}$, key $K_{1}$ and $K_{2}$, and value V are obtained. The query differential attention operation is calculated using the following formula:

(8) $$DiffAttn\left(X\right)=(softmax\left(\frac{Q_{1}K_{1}^{T}}{\sqrt{d}}\right)-\lambda softmax\left(\frac{Q_{2}K_{2}^{T}}{\sqrt{d}}\right))V\;$$

where $W_{i}^{Q}$, $W_{i}^{K}$, and $W_{i}^{V}$ are the projection matrices for each attention head and i∈[1,h] represents the number of differential multi-head attention heads, while λ is a learnable scalar used to control the balance between the two softmax functions.

Next, root mean square layer normalization (RMSNorm) is applied to the output of each attention head, using a fixed multiplier $1-\lambda_{\mathrm{init}}$ as the normalization scaling factor LN(·). The outputs of multiple heads are concatenated and linearly transformed through a learnable projection matrix $W^{O}$ to obtain the output result of the differential multi-head attention $M_{att}^{R}$, as shown in the following formulas:

(9) $$\tilde{M_{l}^{R}}=\left(1-\lambda_{\mathrm{init}}\right)\cdot LN\left(DiffAttn\left(X_{R},W_{i}^{Q},W_{i}^{K},W_{i}^{V},\lambda \right)\right)$$

(10) $$M_{att}^{R}=Concat\left(\tilde{M_{1}^{R}},\cdots ,\tilde{M_{h}^{R}}\right)W^{O}$$

where $\tilde{M_{l}^{R}}$ represents an attention head representation on the input $X_{R}$ after differential attention calculation and normalization for the ith attention head. $\left[\tilde{M_{1}^{R}},\cdots ,\tilde{M_{h}^{R}}\right]$ denotes the concatenated output result of *h* attention heads, and $\lambda_{\mathrm{init}}$ is a hyperparameter used to initialize λ.

Finally, the output of the multi-head attention $M_{att}^{R}$ is fed into a feedforward neural network to obtain the semantic representation of the resumes $M_{R}$:

(11) $$M_{R}=feedforword\left(M_{att}^{R}\right)$$

The differential attention semantic encoding method for the recruitment requirements $M_{P}$ is similar to the above semantic encoding method for resumes.

#### 3.3.2. Self-Attention Mechanism-Based Semantic Encoding for Resumes and Job Positions

The self-attention mechanism dynamically allocates weights to capture the global dependencies inherent in job seekers’ resumes and job requirements, thereby further excavating the semantic structures among skills and enhancing the representation of key features.

Taking job seekers’ resumes as an example, the resumes’ semantic encoding results $M_{R}$ using the differential attention mechanism are used as input, where the resumes’ semantic representation $M_{R}$, where $M_{R}=\left[M_{R}^{1},M_{R}^{2},\ldots ,M_{R}^{l}\right]$, consists of a sequence of semantic representations of *l* skills in the resumes. Let $M_{R}^{l}$ represent the semantic representation of the *l*th skill.

The softmax function is used to calculate the attention scores α, and the formulas are as follows:

(12) $$e_{l}^{R}=v_{\alpha }^{T}tanh\left(W_{\alpha }M_{R}^{l}+b_{\alpha }\right)$$

(13) $$\alpha_{l}=\frac{exp(e_{l}^{R})}{\sum_{l=1}^{n}exp\left(e_{l}^{R}\right)}$$

Here, $V_{\alpha }$ and $W_{\alpha }$ are learnable parameters during training, $b_{\alpha }$ is the bias, Tanh represents the hyperbolic tangent activation function, and $e_{l}^{R}$ indicates the importance of the *l*th skill in the resumes.

Finally, the resumes’ representations of the job seekers are generated by calculating $S^{R}$:

(14) $$S^{R}=\sum_{l=1}^{n}\alpha_{l}M_{R}^{l}$$

The semantic encoding $S^{P}$ for recruitment requirements is similar to the above semantic encoding method for job seekers’ resumes.

### 3.4. Person-Job Fit

#### 3.4.1. Fine-Grained Semantics

In person-job matching tasks, cosine similarity is frequently employed to gauge the similarity between job seekers’ resumes and job requirements. However, relying solely on cosine similarity overlooks the fine-grained semantic matching between job seekers’ resumes and job requirements. To address this, we further introduce dot product similarity in addition to cosine similarity. The following describes the calculation process of fine-grained semantic matching.

First, the resume representation $S^{R}$ of job seekers obtained through hybrid attention mechanism encoding is concatenated with the resume features $S^{R}$ extracted by the GATs to obtain the fused resume representation $V_{r}$. The semantics representation $V_{j}$ of job requirements is similar to that of job seekers’ resumes. The calculation formulas for the resume representation $V_{r}$ and job requirements $V_{j}$ are as follows:

(15) $$V_{r}=concat\left(\left[S^{R};R^{\prime }\right]\right)$$

(16) $$V_{j}=concat\left(\left[S^{P};P^{\prime }\right]\right)$$

Second, cosine similarity is used to calculate the overall semantic similarity $S_{1}$ between the resumes and job requirements. Then, the dot product function is used to individually calculate the similarity $S_{2}$ of key features such as skills and work experience in the resumes and job requirements. After that, the fine-grained semantic similarity obtained by $S_{2}$ is used to supplement the overall similarity $S_{1}$. The $S_{1}$ and $S_{2}$ features are concatenated to obtain a fine-grained semantic feature vector $m_{1}$, which captures the fine-grained differences between the work experience in the job seeker’s resumes and the business needs in the job requirements. The formulas for this are as follows:

(17) $$S_{1}=cosine\left(V_{r},V_{j}\right)\cdot W_{1}$$

(18) $$S_{2}=Dot\left(V_{r},V_{j}\right)\cdot W_{2}$$

(19) $$m_{1}=concat\left(\left[S_{1};S_{2}\right]\right)$$

where $W_{1}$ and $W_{2}$ are trainable weight matrices.

Vector $m_{1}$ primarily focuses on the fine-grained directional differences in the representation vectors of job seekers’ resumes and job requirements while being insensitive to distance. A more effective approach is to compute the bilinear distance $m_{2}$ between resumes and job requirements based on vector distances. The bilinear distance $m_{2}$ is calculated as follows:

(20) $$m_{2}=Bilinear\left(V_{r},V_{j}\right)$$

where Bilinear denotes the bilinear function.

The aforementioned vector representations measure the alignment between resumes and job requirements from the perspectives of fine-grained semantics and bilinear distance semantics, yet they neglect the interactive relationships at the feature level. To this end, we construct an interaction vector $m_{3}$. By combining the representation vectors of resumes and job positions, we calculate the element-wise absolute differences, thereby achieving fine-grained semantic interactions at the level of resume-job position pairs. Finally, the resume representation $V_{r}$, job requirements representation $V_{j}$, and absolute value of the difference $\left|V_{r}-V_{j}\right|$ between these two vectors are concatenated to obtain $m_{3}$, and the calculation formula is as follows:

(21) $$m_{3}=concat\left(\left[V_{r};V_{j};\left|V_{r}-V_{j}\right|\right]\right)$$

#### 3.4.2. Enhanced Feature Fusion

The multilayer perceptron (MLP) has limited ability to capture the nonlinear relationships between job seekers’ resumes and job requirements. Therefore, the feature fusion strategy of the KAN [27] is introduced to more effectively capture the nonlinear relationships between job seekers’ work experience and job requirements.

A KAN comprising *L* layers adopts a form of multi-layer composite functions, and its computational formula is as follows:

(22) $$KANLinear\left(x\right)=\left(\Phi_{L-1}\circ \Phi_{L-2}\circ \cdots \circ \Phi_{1}\circ \Phi_{0}\right)\left(x\right)$$

Here, $\Phi_{l}$ denotes the activation function matrix, where each activation function is constructed from a learnable parameterized spline curve, which is calculated as follows:

(23) Φ(x)=w·(b(x)+spline(x))

(24) $$b\left(x\right)=\frac{x}{1+e^{-x}}$$

(25) $$spline\left(x\right)=\sum_{i}c_{i}B_{i}\left(x\right)$$

where Φ(x) represents the activation function, *w* is the trainable weight matrix, *b*(*x*) is the basis function, and *spline*(*x*) is a nonlinear function formed by the linear combination of B spline basis functions, while $c_{i}$ consists of trainable parameters and $B_{i}\left(x\right)$ is the *i*th B spline basis function. To ensure the expressiveness and smoothness of the activation functions, in the experiments, the order of the B spline functions was set to three (i.e., cubic B splines), and the grid size for spline interpolation was set to five.

In the person-job fit task, the KAN enhances feature fusion by dynamically adjusting the learning strategy for B spline curves. It integrates the fine-grained semantic feature vectors $m_{1}$ of resumes and job positions, the bilinear distance vectors $m_{2}$ between resumes and job positions, and the fine-grained semantic interaction vectors $m_{3}$ at the resume-job position level. This integration yields the final matching results *y* between resumes and job positions:

(26) $$y=KANLinear(\left[m_{1};m_{2};m_{3}\right])$$

The cross-entropy loss (CEL) function is a commonly employed loss function in the training process of neural networks, serving to measure the discrepancy between the predicted values and true values. During the model training phase, the CEL guides the model to continuously optimize network parameters, reduce the output loss, and enhance the performance of person-job fitting. Finally, the CEL function is used to train the FSEGNN-PJF framework. The CEL is calculated as follows:

(27) $$L_{CEL}=-\left(S^{g}\left(G_{y},G_{l}\right)log\left(S_{y,l}\right)+\left(1-S^{g}\left(G_{y},G_{l}\right)\right)log\left(1-S_{y,l}\right)\right)$$

where *g* represents the number of batches in the training process, $G_{y}$ denotes the predicted value, $G_{l}$ denotes the true value, $S^{g}\left(G_{y},G_{l}\right)$ is the predicted similarity value, and $S_{y,l}$ represents the true similarity value between $G_{y}$ and $G_{l}$.

## 4. Experiments

In this section, the experimental set-up, baselines, and evaluation metrics are introduced first. Then, the performances of the proposed FSEGNN-PJF framework and baselines are reported. Finally, an ablation study and case study are designed to demonstrate the effectiveness of the proposed components and the interesting characteristics of the FSEGNN-PJF framework.

### 4.1. Dataset

All of the records in a real-world recruitment dataset were anonymized by removing user profiles. The experimental dataset included information on recruitment job positions, job seekers’ resumes, and the matching label for recruitment results, consisting of 4278 samples. The first job position column represents the recruitment requirements for the job position, the second resume column describes the experiences in the job seeker’s resume (work experience, etc.), and the third matching label column indicates the label for the recruitment result. Essentially, the person-job fit task is a binary classification problem. If the recruitment is successful, then the label is 1, and if unsuccessful, then the label is 0.

### 4.2. Experimental Set-Up

All experiments were conducted on a computer equipped with a 12th Gen Intel(R) Core(TM) i7-12700H processor, 16.0 GB of onboard RAM, and an NVIDIA GeForce RTX 3060 Laptop GPU.

In the experiments, the resumes and job requirements were initialized using the Word2Vec word embedding method, with a word embedding dimension of 128. The co-occurrence window size was set to 2. The hidden layer size of the GATs was 128. The number of differential transformer layers was 2, the number of attention heads in the differential multi-head attention mechanism was 8, the dimension of the differential transformer feedforward layer was 2048, and the weight coefficient lambda value in the differential loss function was 0.8. The AdamW optimizer was chosen to optimize the framework, with a training batch size of 128 and a learning rate of 0.0005. Table 1 shows the parameter settings for the experiments.

### 4.3. Baselines

To validate the efficacy of the FSEGNN-PJF framework, comprehensive comparisons were conducted with three methodological categories: conventional supervised learning approaches, deep learning methods, and state-of-the-art person-job matching models. All methods can be described as follows:

- **Conventional supervised learning methods** include decision tree (DT), support vector machine (SVM), Adaboost (AB), and gradient boosting decision tree (GBDT), implemented using Doc2Vec vector as feature inputs.
- **DSSM** utilizes dual deep neural networks for semantic vector projection with cosine similarity measurement [18].
- **Siamese-LSTM** involves inputting two sentences into the same LSTM model to obtain forward and backward sentence vectors and finally inputting the sentence pair representations into the softmax layer [28].
- **ABCNN** implements attention-based convolutional neural networks for sentence pair modeling [29].
- **MatchPyramid** has sentence pairs inputted, features extracted via a CNN, and the matching scores finally outputted using an MLP [30].
- **PJFNN** involves taking resume and job requirement pairs as input, encoding resume and job requirement texts by employing a parallel CNN, and calculating the matching results by using the cosine similarity [4].
- **BPJFNN** [1] is a simplified version of APJFNN. It takes resumes and recruitment requirements as the input sequences and employs BiLSTM to learn the semantic representation of the job seekers’ resumes and job requirements.
- **APJFNN** considers resumes and job requirements as input sequences, uses BiLSTM to encode job seekers’ resumes and job requirements, and adopts hierarchical ability-aware attention mechanisms to learn word-level semantic representations of resumes and job requirements [1].
- **IPJF** uses a CNN and collaborative attention mechanism to represent resumes and job requirements, and it employs an MLP to predict the matching between resumes and job positions [31].
- **conSultantBERT** employs fine-tuned Siamese sentence-bert to match job seekers and jobs [32].
- **MKPM** combines BiLSTM encoding with attention mechanism for keyword pairs extracted and interaction feature generation [24].
- **InEXIT** leverages BERT and a multi-head attention mechanism for encoding and cross-attribute interaction modeling and uses an aggregation matching layer to predict the matching between resumes and job positions [5].
- **FSEGNN-PJF** is the proposed framework in this paper.

### 4.4. Evaluation Metrics

The binary classification nature of the person-job fit necessitates four evaluation metrics: accuracy, precision, recall, F1 score (F1), and AUC. Accuracy refers to the proportion of correctly predicted positive (successful hiring) and negative (unsuccessful hiring) samples to the total number of samples. Precision is defined as the proportion of correctly predicted successful hiring samples among all samples predicted as successful hirings. Recall is the proportion of correctly predicted successful hiring samples to all actual successful hiring samples. The F1 score is the harmonic mean of precision and recall. AUC is the area under an ROC curve. The ROC curve is determined by the true positive rate (TPR) and false positive rate (FPR). These metrics collectively assess model performance. The formulas for these evaluation metrics are as follows:

(28) $$\mathrm{accuracy}\;=\;\frac{\mathrm{TP}\;+\;\mathrm{TN}}{\mathrm{TP}\;+\;\mathrm{FN}\;+\;\mathrm{FP}\;+\;\mathrm{TN}}$$

(29) $$\mathrm{precision}\;=\;\frac{\mathrm{TP}}{\mathrm{TP}\;+\;\mathrm{FP}}$$

(30) $$\mathrm{recall}\;=\;\frac{\mathrm{TP}}{\mathrm{TP}\;+\;\mathrm{FN}}$$

(31) $$F1\;=\;\frac{2\times \mathrm{precision}\times \mathrm{recall}}{\mathrm{precision}\;+\;\mathrm{recall}}$$

(32) $$\mathrm{TPR}\;=\;\frac{\mathrm{TP}}{\mathrm{TP}\;+\;\mathrm{FN}}$$

(33) $$\mathrm{FPR}\;=\;\frac{\mathrm{FP}}{\mathrm{FP}\;+\;\mathrm{TN}}$$

Here, true positive (TP) represents the number of samples that are actual successful hirings and are also predicted to be successful hirings. True negative (TN) represents the number of samples that are actually unsuccessful hirings and are also predicted to be unsuccessful hirings. False negative (FN) represents the number of samples that are actually successful hirings but are predicted to be unsuccessful hirings. False positive (FP) represents the number of samples that are actually unsuccessful hirings but are predicted to be successful hirings.

The accuracy, precision, recall, F1 score, and AUC values ranged from 0 to 1. The higher the value, the better the performance of the method.

### 4.5. Overall Performance

To verify the effectiveness of the proposed FSEGNN-PJF framework on real recruitment datasets, the FSEGNN-PJF framework was compared with several baselines. Table 2 lists the performance of the FSEGNN-PJF framework and baselines in terms of accuracy, precision, recall, F1 score, and AUC. The first column shows the 14 baselines for comparison and the proposed method. ${\mathrm{FSEGNN-PJF}}_{transformer}$ refers to the method proposed in this paper, which utilizes the traditional transformer. FSEGNN-PJF is the final framework selected in this paper, which employs the differential transformer. Columns 2–6 show the experimental results for the accuracy, precision, recall, F1 score, and AUC. The optimal results for each evaluation metric are shown in bold.

As can be observed from Table 2, among all the baselines, the classic supervised learning methods exhibited the worst overall performance. For the classic supervised learning methods, in terms of accuracy, the AB model performed the worst, while the GBDT model performed the best.

Compared with the classic supervised learning methods, the deep matching models demonstrated superior overall performance in person-job matching tasks. Among them, the classic deep semantic matching model DSSM exhibited the poorest performance among all deep matching models. However, the performance of DSSM was significantly better than that of GBDT, the best-performing model among the classic supervised learning methods. In terms of accuracy, DSSM’s was 16.97% higher than GBDT’s. In terms of F1 score, DSSM showed an improvement of 13.94% over GBDT. This may be due to the fact that GBDT relies on text feature embeddings generated by the Doc2Vec method, which results in feature vectors containing more noise and difficulty in effectively capturing subtle semantic differences between job seekers’ resumes and job requirements.

Among deep matching models, MKPM demonstrated further improved performance compared with DSSM. This performance improvement may be attributed to the fact that MKPM can more accurately identify matching relationships when associating job seekers’ resumes with job requirements, whereas the DNN structure of DSSM cannot fully reflect the matching degree between resumes and jobs when dealing with complex matching relationships.

Driven by the demand for complex person-job matching relationships, the APJFNN model, designed for person-job matching tasks, demonstrated improvements of 1.63% in accuracy and 1.25% in F1 score compared with MKPM. The experimental results indicate that the APJFNN model has advantages in modeling person-job matching interaction information.

Further observation of the experimental results revealed that the pretrained model conSultantBERT performed better than the APJFNN model. This suggests that BERT-based person-job matching methods can effectively capture the semantics of job seekers’ resumes and job requirements, indicating that the BERT method is beneficial for modeling person-job matching tasks.

It is gratifying to find that, in terms of accuracy and F1 score, the performance of the ${\mathrm{FSEGNN-PJF}}_{transformer}$ method was superior to all baselines in person-job matching tasks. The experimental results for accuracy, precision, recall, F1 score, and AUC were 0.9182, 0.9220, 0.9178, 0.9199, and 0.9203, respectively. The experimental results demonstrate the effectiveness of the ${\mathrm{FSEGNN-PJF}}_{transformer}$ method in capturing deep semantic and structural information in resumes and job titles.

The experimental results of the FSEGNN-PJF framework on the accuracy, precision, recall, F1 score, and AUC evaluation metrics were 0.9369, 0.9486, 0.9269, 0.9376, and 0.9248, respectively. The results indicate that the performance of FSEGNN-PJF was superior to all baselines. Compared with the ${\mathrm{FSEGNN-PJF}}_{transformer}$ method, the performance of the FSEGNN-PJF framework in terms of the accuracy, precision, recall, and F1 score evaluation metrics improved by 1.87%, 2.66%, 0.91%, and 1.77%, respectively. This phenomenon suggests that the differential transformer in the FSEGNN-PJF framework reduces invalid noise, enabling the method to focus on the matching between job seekers’ core skills and job requirements when dealing with complex person-job matching scenarios, which is conducive to achieving a precise person-job fit.

### 4.6. Ablation Study

To evaluate the impact of each variant on the performance of the FSEGNN-PJF framework, ablation experiments were conducted. Five variants of the FSEGNN-PJF framework were designed: (1) FSEGNN-PJF w/o KAN denotes that a KAN was not used, and it adopted an MLP instead of a KAN to fuse features; (2) FSEGNN-PJF w/o GAT denotes the removal of the GAT variant, i.e., removing the graph-based resume-job semantic encoding; (3) FSEGNN-PJF w/o D-Transformer denotes the removal of the D-Transformer variant, i.e., removing the differential transformer; (4) FSEGNN-PJF w/o FGSM denotes the removal of the fine-grained semantic variant; and (5) FSEGNN-PJF w/o Satt denotes the removal of the self-attention variant. The results of the ablation experiments are shown in Table 3.

The experimental results of the ablation study show the following:

(1) Regardless of which variant was removed, the performance decreased, indicating that each variant was useful in the overall framework.
(2) The FSEGNN-PJF w/o KAN variant experienced decreases in accuracy, precision, recall, and F1 score of 2.57%, 3.54%, 1.37%, and 2.44%, respectively. The performance degradation of the proposed framework when using an MLP for feature fusion indicated the insufficiency of the MLP in modeling the nonlinear relationships among job seekers’ experiences, skills, and job requirements. The results indicate that the KAN could effectively capture multi-dimensional complex nonlinear features between job seekers’ experience, skills, and job requirements.
(3) The FSEGNN-PJF w/o GAT variant showed decreases in accuracy, precision, recall, and F1 score of 2.10%, 1.08%, 3.19%, and 2.17%, respectively. The results show that the resume-job semantic encoding module of the graph attention network could effectively capture deep semantic associations and structural information between resumes and job requirements.
(4) The FSEGNN-PJF w/o D-Transformer variant demonstrated decreases in accuracy and F1 score of 2.80% and 2.52%, respectively. The results indicate that the D-Transformer variant could reduce data noise and allow the frame to focus more on the matching between job seekers’ core skills and job requirements.
(5) The performance degradation of the FSEGNN-PJF w/o FGSM variant demonstrates that this module can effectively capture the fine-grained semantics of job seekers’ resumes and job requirements.
(6) The FSEGNN-PJF w/o Satt variant exhibited decreases of 7.71%, 6.82%, 8.67%, 7.78%, and 6.45% in accuracy, precision, recall, F1 score, and AUC, respectively. These results demonstrate the critical role of the self-attention mechanism in capturing intra-sentential semantic dependencies within resumes and job descriptions, as well as perceiving key features such as skills.

### 4.7. Robustness to Noise

To validate the robustness of the proposed framework against input noise in real-world recruitment application scenarios, three common types of noise (e.g., word deletion, spelling errors, and word repetition) were simulated in experiments. Specifically, word deletion was achieved by randomly removing some words to simulate situations where job seekers or recruiters provide incomplete or disorganized inputs. Spelling errors were simulated by substituting characters or words to mimic typing mistakes or non-standard spellings. Word repetition was simulated by randomly duplicating and inserting existing words to represent redundant expressions or repeated information caused by copy-pasting. These noise perturbations simulated common spelling errors or formatting issues in job seekers’ resumes or job descriptions. Artificial noise was introduced into the original test dataset, and the performance of the proposed framework was evaluated under the same settings. The experimental results for robustness, with the highest scores displayed in bold font, are shown in Table 4.

As can be seen from the table above, the framework demonstrated the best overall performance across various evaluation metrics on the original data. After introducing noise, there was a slight decline in performance, but it remained generally stable. Word deletion led to a relatively more pronounced performance drop, possibly because the randomly removed words contained information expressing core semantics or key entities, resulting in incomplete semantic representation. In contrast, the impact of word repetition noise was relatively minor, indicating that the framework has a certain degree of robustness against redundant information. The impact of spelling error noise was the smallest, suggesting that the proposed framework possesses a certain spelling error tolerance capability.

Under the three types of noise perturbations, the proposed framework’s performance remained relatively stable, with the F1 score consistently maintained at about 92%. The experimental results demonstrate that the FSEGNN-PJF framework exhibits good robustness in real-world scenarios.

### 4.8. Case Study

The attention mechanism helps enhance the interpretation ability of person-job matching tasks. In this subsection, the experimental results will be illustrated through visualization of the attention results.

**Figure 2:**
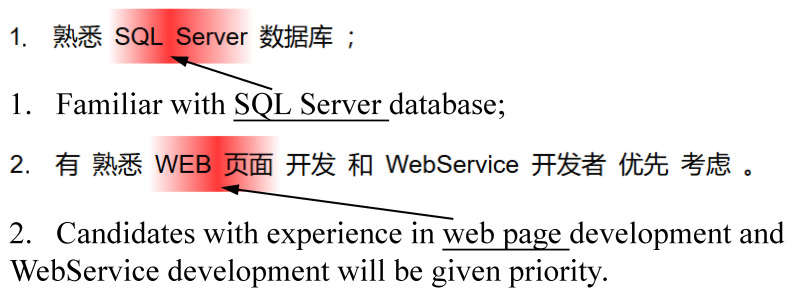
Ability-aware examples.

The attention mechanism can intuitively display the importance of different words in job posting requirements, as shown in Figure 2. In Sentence 1, some words are highlighted as key phrases, such as “SQL Server”. The highlighted key phrases are shown in dark red, indicating that the attention weight of this key phrase is higher. This experimental results indicate that the proposed FSEGNN-PJF framework paid a great deal of attention to job skills. In Sentence 2, the skill of “WEB page” is highlighted as a key phrase. In contrast, some more general or common words, such as “WebService development”, are not focused on. Analysis of the experimental results shows that the proposed FSEGNN-PJF framework in this paper performs well in capturing key skills.

Through the visualization results of the attention mechanism, it can be clearly seen that higher weights were assigned to the key skills that were focused on. This case study demonstrates that the proposed FSEGNN-PJF framework can provide a good interpretation ability for person-job fit tasks.

## 5. Conclusions

This paper proposes a novel fine-grained semantics-enhanced graph neural network for person-job fit (FSEGNN-PJF) framework. Specifically, given a pair of resumes and job positions, first, a GAT was used to semantically encode the job seekers’ resumes and job requirements. Second, a hybrid attention mechanism was employed to semantically encode the job seekers’ resumes and job requirements. Third, a fine-grained semantic matching calculation method was designed to deeply fuse the semantic features of the job seekers’ resumes and job requirements using an enhanced feature fusion strategy. Finally, the experimental results validated the effectiveness and robustness of the proposed FSEGNN-PJF framework.

In the future, we will explore person-job fit tasks oriented toward multimodal data (e.g., audio or video data from interviews). Further research will be conducted on other text representation methods for resumes and job requirements to improve the performance of person-job fitting.

## Figures and Tables

**Table 1:** Parameter settings.

Parameter	Value
Word2Vec word embedding dimension	128
Co-occurrence window size	2
GAT hidden layer size	128
DIFF Transformer layers	2
DIFF Transformer heads	8
DIFF Transformer FFN dimension	2048
Weighting factor lambda	0.8
Optimizer	AdamW
Batch size	128
Learning rate	0.0005

**Table 2:** Overall performance of FSEGNN-PJF framework and all baselines for person-job fit in terms of accuracy, precision, recall, F1 score, and AUC.

Model	Accuracy	Precision	Recall	F1	AUC
DT	0.5541	0.5561	0.5458	0.5509	0.5456
SVM	0.5327	0.5430	0.4268	0.4779	0.5515
AB	0.5304	0.5264	0.5224	0.5244	0.5227
GBDT	0.5710	0.5617	0.5956	0.5782	0.5304
DSSM	0.7407	0.7121	0.7231	0.7176	0.8088
Siamese-LSTM	0.7523	0.7542	0.7400	0.7470	0.7504
ABCNN	0.8341	0.8333	0.8314	0.8323	0.8384
MatchPyramid	0.7620	0.7530	0.7790	0.7658	0.7640
PJFNN	0.8598	0.8230	0.8821	0.8515	0.8668
BPJFNN	0.8668	0.8594	0.8462	0.8527	0.8526
APJFNN	0.8738	0.8524	0.9203	0.8850	0.8781
IPJF	0.8691	0.8899	0.8584	0.8739	0.8519
conSultantBERT	0.8855	0.8971	0.8767	0.8868	0.8925
MKPM	0.8575	0.8323	0.9169	0.8726	0.8996
InEXIT	0.8528	0.8714	0.8356	0.8531	0.8909
${\mathrm{FSEGNN-PJF}}_{transformer}$	0.9182	0.9220	0.9178	0.9199	0.9203
FSEGNN-PJF	**0.9369**	**0.9486**	**0.9269**	**0.9376**	**0.9248**

**Table 3:** Ablation study on FSEGNN-PJF framework with various modules for person-job fit.

Model	Accuracy	Precision	Recall	F1	AUC
FSEGNN-PJF	0.9369	0.9486	0.9269	0.9376	0.9248
FSEGNN-PJF w/o KAN	0.9112	0.9132	0.9132	0.9132	0.9046
FSEGNN-PJF w/o GAT	0.9159	0.9378	0.8950	0.9159	0.9095
FSEGNN-PJF w/o D-Transformer	0.9089	0.8982	0.9269	0.9124	0.8837
FSEGNN-PJF w/o FGSM	0.9206	0.9302	0.9132	0.9217	0.8905
FSEGNN-PJF w/o Satt	0.8598	0.8804	0.8402	0.8598	0.8603

**Table 4:** Performance of the FSEGNN-PJF framework on the test sets and perturbed samples of the person-job fit task.

Dataset	Accuracy	Precision	Recall	F1	AUC
Original data	**0.9369**	**0.9486**	**0.9269**	**0.9376**	**0.9278**
Deletion	0.9206	0.9302	0.9132	0.9217	0.9207
Spelling errors	0.9252	0.9269	0.9269	0.9269	0.9252
Repeat	0.9252	0.9309	0.9224	0.9266	0.9253

## Data Availability

The data used to support the findings of this study are available from the corresponding author upon request.
